# A Retrospective Analysis of Pattern of Mental Health Problems in COVID-19 Patients in a Tertiary Care Hospital in India

**DOI:** 10.1192/bjo.2022.182

**Published:** 2022-06-20

**Authors:** Jwalamukhi Chidambaram Thirugnanam, Ringhoo Theresa Jose

**Affiliations:** Lourdes Hospital, Ernakulam, India

## Abstract

**Aims:**

A. To investigate the nature of liaison psychiatry consultations for COVID-19 patients in a tertiary care hospital in India B. To assess pattern and prevalence of mental health disorders and management, in COVID-19 patients in a tertiary care hospital in India.

**Methods:**

Retrospective chart-based study

Data from medical records of 1600 confirmed COVID-19 patients was studied and charts of 368 patients among that who, during their in-patient stay for COVID-19 treatment in Lourdes Hospital, Kochi (September 2020 - December 2021), received liaison psychiatry consultation was selected for retrospective analysis

**Results:**

Psychiatric consultations were sought for 23%(368) patients with COVID-19 (1600) during the study period. The most common symptoms of mental health problems for referral were sleep disturbance (74.9%), agitation/restlessness, increased tension (50.3%), depressive symptoms like low mood, loss of interest (11.1%) and psychotic symptoms like talking to self, hearing voices, suspiciousness (8%). Liaison psychiatry consultation was most sought-after for critically ill patients (69.2%), with disturbed behaviour as the most common presenting complaint. Psychiatric diagnoses included in the spectrum of delirium (39.3%), sleep disorders (33.3%), anxiety (15.5%), depression (7.1%) and psychosis (4.8%). In terms of psychiatric treatments, 95.9% of patients who received psychiatric consultation were treated with psychotropic medications, including non-benzodiazepine sedative-hypnotic agents (54.8%), anti-psychotic (26.2%), benzodiazepines (22.6%) and antidepressant (10.7%). The symptoms of 61% of patients had improved and they were prescribed medications to continue the treatment on discharge.

**Conclusion:**

A significant proportion of hospitalized COVID-19 patients experienced mental health problems, especially patients in intensive care unit. Data that emerged from this study regarding pattern of mental illness and management options will serve as a template for psychiatrists to liaise with medical teams to treat future patients.

